# Golden Jubilee, 2008

**DOI:** 10.4103/0019-5545.33250

**Published:** 2007

**Authors:** T. S. Sathyanarayana Rao

**Affiliations:** Department of Psychiatry, JSS Medical College and Hospital, Mysore - 570 004, India

2008 is the golden jubilee year for the journal. Though it took birth in 1949 as “Journal of Neurology and Psychiatry” under the editorship of Dr. N. N. De, officially it became “Indian Journal of Psychiatry” in 1958 during the editorship of Dr. L. P. Verma. It is a proud moment for our society on the one hand - as IJP has served as a mouthpiece holding aloft the flag of our society - its mind, attitude and character! It is also a time for introspection to harness our energy to reach much greater height! There is no dearth of talent. It has been handled by stalwarts who have left the mark - individually, professionally and socially! The names of the distinguished past editors reads like who's who of the society and our reverence is due for them. We mentioned in great detail the proposed changes for our journal and many aspects are in place.[1] The website and online editorial processes - submission, review and publication-are getting strengthened. The inauguration of the new website www.indianjpsychiatry.org was made by our distinguished president Dr. Indla Ramasubba Reddy on April 15, 2007 at Kolkata, where in addition many things were proposed and approved at the EC meeting. I am placing the points discussed there and certain new suggestions received in due course-for widest dissemination to garner as many new ideas as possible!

Some of the proposals for the golden jubilee celebration of the journal are entered here for your kind attention, thoughts and suggestions:

1. To archive all the past journals since 1958 at our website which will be available for viewing and free downloading to all the members. Is anybody having the issues between 1949 to 1958? Effort is being made to trace out all the issues to convert them for electronic viewing and uploading. Please watch this column for the progress made during the next issue of IJP 49(3) due for release in July 2007.
2. To bring out a commemorative volume during ANCIPS-2008 at Kolkata, so also a special issue of past presidential addresses.
3. Articles by the past editors who are around and to publish memorial articles on other deceased past editors by their associates.
4. We are going to have a special session and an exhibition for IJP at ANCIPS-2008. I request you to spare rare photographs of past editors, group photos, journal-related events, copies of the very old journals etc. Your contributions will be duly acknowledged.
5. I am inviting the articles on our past editors-providing a brief sketch of them, their work and life. All the write-ups are welcome which may be edited keeping in mind the editorial policy. Also I am requesting articles it has most influences in the IJP down the ages, historical land marks in the development of IJP including little events such as the year in which the supplement started, the year in which the publication of the subject index comment so also any background stories and anecdotes concerning our journal, if possible, with photographs.
6. The work has already started. I welcome your involvement and contribution to make the golden jubilee celebration of the journal a memorable one. My wish is to involve each and every member in this noble and much needed work and I am confident of your association, help and guidance.
7. Any suggestions to increase the frequency of the journal? Any ideas?

All ideas however great or small are welcome! Call me on my mobile, send your ideas, views and suggestions at editor@indianjpsychiatry.org or personally at tssrao19@gmail.com or tssrao19@yahoo.com.

In the meantime, the supplement of IJP should have reached by now. This issue should be in your hand before June. 49(3) is scheduled for release in July 2007 with a special feature on U.G. Psychiatric education. Happy reading!

## References

[1] Sathyanarayana Rao TS (2007). An opportunity and a beginning. Indian J Psychiatry.

